# Myocarditis monitoring during clozapine initiation: audit of practice in an NHS trust

**DOI:** 10.1192/bjo.2026.12079

**Published:** 2026-08-25

**Authors:** Madelaine Bridges, Agostina Secchi, Yash Kumar, Von-de viel Nettey, Jagdip Bahia, Eromona Whiskey, Sukhi S. Shergill

**Affiliations:** Kent and Medway NHS and Social Care Partnership Trust, Maidstone, UK; Pharmacy, Kent and Medway NHS and Social Care Partnership Trust, Maidstone, UK; Medway School of Pharmacy, University of Kent, UK; Pharmacy Department, South London and Maudsley NHS Foundation Trust, London, UK; Institute of Pharmaceutical Science, King’s College London, UK; Psychosis Studies, Institute of Psychiatry, Psychology & Neuroscience, King’s College London, UK; Kent and Medway Medical School, Canterbury, UK

**Keywords:** Clozapine, myocarditis, troponin, C-reactive protein

## Abstract

**Background:**

Clozapine is the most effective treatment for individuals with treatment-resistant schizophrenia, but concerns about adverse effects continue to limit its use. In contrast to haematological monitoring, which is governed by established protocols, there is no standardised framework for detection of clozapine-associated myocarditis. Clozapine-induced myocarditis is rare but potentially fatal and typically occurs within the first 4 weeks of treatment. As early symptoms such as fever and tachycardia overlap with common side-effects of clozapine, diagnosis is often delayed, and clozapine may be unnecessarily discontinued. Evidence suggests that routine monitoring of troponin and C-reactive protein (CRP), alongside assessment of vital signs and electrocardiography (ECG), can support earlier detection and safer treatment.

**Aims:**

To evaluate cardiac monitoring practice in patients initiating clozapine treatment.

**Method:**

We undertook a retrospective review of all patients registered to start clozapine at Kent and Medway Mental Health National Health Service (NHS) Trust between January 2021 and March 2024. Data were collected on baseline ECG completion, weekly troponin and CRP testing during the first 4 weeks, and documented cardiac symptoms.

**Results:**

Of 219 patients, 180 had complete data. Baseline ECG was completed in 80% of cases. No routine troponin monitoring was identified, and CRP testing was infrequent. Tachycardia occurred in more than 40% of patients. Fifteen patients were referred to cardiology before initiation, as well as six in the first 4 weeks and five thereafter. Twelve patients (7%) discontinued clozapine early, mainly owing to tachycardia.

**Conclusions:**

The findings indicate an absence of systematic myocarditis monitoring. Incorporating weekly troponin and CRP testing into existing mandatory blood testing could improve safety, guide appropriate cardiology referrals and reduce unnecessary discontinuation.

Treatment-resistant schizophrenia is a severe and debilitating condition characterised by inadequate response to at least two antipsychotic medications at therapeutic doses. Clozapine, which remains the gold standard and the only antipsychotic licensed for treatment-resistant schizophrenia, offers superior efficacy in reducing symptoms and improving functional outcomes compared with other agents.^1,2^ Despite this, clozapine remains underutilised worldwide, largely owing to concerns about its complex safety profile and clinicians’ confidence in managing its adverse effects.^3,4^

The haematological risk of agranulocytosis associated with clozapine is well recognised and can be mitigated through internationally standardised monitoring protocols. By contrast, clozapine-induced myocarditis, a rare but potentially life-threatening adverse effect, receives less attention.^5,6^ Clozapine-induced myocarditis typically occurs within the first 4 weeks of treatment initiation and is characterised by inflammation of the myocardium.^6,7^ The exact mechanism of clozapine-induced myocarditis is not fully understood but is likely to be multifactorial, involving pro-inflammatory cytokine release, catecholaminergic activation, oxidative stress, and cardiomyocyte injury through apoptosis and ischaemic processes. ^8,9^

Although the reported absolute fatality risk is very low (0.0004), the condition can lead to significant morbidity if not detected early.^10^ Prevalence estimates vary widely, ranging from 0.03% to 8%; ^11,12^ the highest rates have been reported in Australian cohorts, probably owing to detection and reporting biases within these populations. This high variability is likely to reflect differences in diagnostic criteria, monitoring practices and reporting standards. Nonetheless, it is important to emphasise that the risks of myocarditis and cardiomyopathy are low and should not preclude a trial of clozapine in individuals with treatment-resistant schizophrenia.

Early detection of clozapine-induced myocarditis is challenging because it may be asymptomatic or present with non-specific symptoms such as fever, tachycardia, fatigue and palpitations – features that can be misattributed as common clozapine side-effects. Some of these side-effects can occur in up to 40–50% of patients within the first month of treatment during the titration phase. Evidence suggests that this is linked to immunomodulatory actions of clozapine driven by increased levels of cytokines. The high prevalence of these side-effects means that distinguishing between symptoms of myocarditis and the common immunomodulatory effects of clozapine is complex.^13^ Current evidence supports the use of cardiac biomarkers, particularly troponin and C-reactive protein (CRP), in combination with electrocardiography (ECG), as effective tools for identification of early myocardial inflammation.^14–16^ The rate of detection of suspected or definite cases of clozapine-induced myocarditis is demonstrably higher with systematic monitoring than without and reduces the risk of potentially fatal outcomes.^17^ However, monitoring practices remain inconsistent. For example, according to the clozapine policy of the Kent and Medway Mental Health National Health Service (NHS) Trust (KMMH), troponin and CRP are only measured when cardiac concerns are raised, rather than as part of routine initiation protocols. This reactive approach could be associated with delayed diagnosis and possible unnecessary discontinuation of clozapine, with uncertain but potentially negative implications for treatment outcomes.

Given these challenges, establishing systematic cardiac monitoring during clozapine initiation is critical. Baseline ECG and serial weekly measurements of troponin and CRP during the first 4 weeks could facilitate early detection of clozapine-induced myocarditis without imposing additional monitoring burden,^18^ as these tests can be integrated into mandatory weekly full blood count schedules. However, the extent to which these practices are currently implemented within KMMH is unclear.

## Aims and objectives

In this first trust audit, we aimed to evaluate baseline cardiac monitoring and early surveillance for myocarditis during clozapine initiation across KMMH services. Specifically, the objectives were to:

- assess whether baseline cardiac investigations (ECG, troponin, CRP) were performed before clozapine initiation;
- determine the frequency of weekly troponin and CRP monitoring during the first 4 weeks of treatment;
- review documentation of cardiac-related symptoms (tachycardia, chest pain, fever, breathlessness) during initiation;
- identify the proportion of patients referred to cardiology and those who discontinued clozapine within the first 4 weeks.

### Audit standards

Baseline assessment (before initiation) consisted of ECG and CRP and serum troponin measurements, as well as vital signs (pulse, blood pressure, temperature, respiratory rate). Ongoing monitoring (first 4 weeks of treatment) comprised weekly CRP and troponin measurements (in addition to mandatory full blood count); daily monitoring of pulse, blood pressure and temperature; and clinical assessment of symptoms suggestive of myocarditis (e.g. chest pain, fever, breathlessness).

Action thresholds were as follows. If CRP > 100 mg/L or troponin >2× upper limit of normal, clozapine must be stopped immediately and the patient referred for urgent cardiology review.

These audit standards were derived from Wagner et al^18^ and the recommendations of the Maudsley Prescribing Guidelines.^25^

## Method

We conducted a retrospective audit of clinical records for all patients who initiated clozapine treatment both in in-patient and community settings within KMMH between January 2021 and March 2024. Patient lists were obtained from the Clozapine Patient Monitoring System, one of three national registries responsible for monitoring clozapine use in the United Kingdom.

Patients were included if they were adults aged 18 years and above who initiated clozapine during the study period, regardless of whether treatment was continued or discontinued, and were under the care of KMMH. Those younger than 18 years were excluded, as were those who opted out of data use for research, individuals residing in Kent but managed by other mental health trusts, and those with unavailable clinical notes.

Data extracted included demographic variables (age, gender, ethnicity, diagnosis), treatment details (date of clozapine initiation and discontinuation, if applicable) and cardiac monitoring practices. Specifically, we reviewed progress notes for documentation of baseline ECG and any evidence of underlying cardiovascular disease or symptoms suggestive of myocarditis, including tachycardia, chest pain, shortness of breath, flu-like symptoms and referrals to cardiology. We also recorded whether clozapine was discontinued (temporarily or permanently) and whether discontinuation was related to suspected cardiac events. Laboratory data for troponin and CRP were obtained directly from the local pathology system using secure credentialed access. This approach minimised transcription errors and reduced the risk of missing data, as these results are not consistently documented in progress notes. We collected baseline troponin and CRP values and assessed whether weekly measurements were performed during the first 4 weeks of clozapine initiation.

All data were accessible to the investigators in their capacity as healthcare professionals within KMMH. Ethical approval was not required as the project was classified as a clinical audit, and informed consent was not required as only non-identifiable information was used; approval was granted by the KMMH Clinical Audit and Effectiveness Committee (ref: 5405-24). Data were analysed descriptively using Microsoft Excel 2019 for Windows (version 1808; Microsoft Corporation, Redmond, Washington, USA; https://www.microsoft.com/en/microsoft-365/excel?market=af).

All procedures contributing to this work comply with the ethical standards of the relevant national and institutional committees on human experimentation and with the Helsinki Declaration of 1975, as revised in 2013.

## Results

KMMH serves a local population of approximately 1.8 million people. Relative to other mental health trusts in the UK, its clozapine use is lower than average.^3^ As of January 2026, according to the clozapine registry, 662 patients were prescribed clozapine in the trust.

**Fig. 1:**
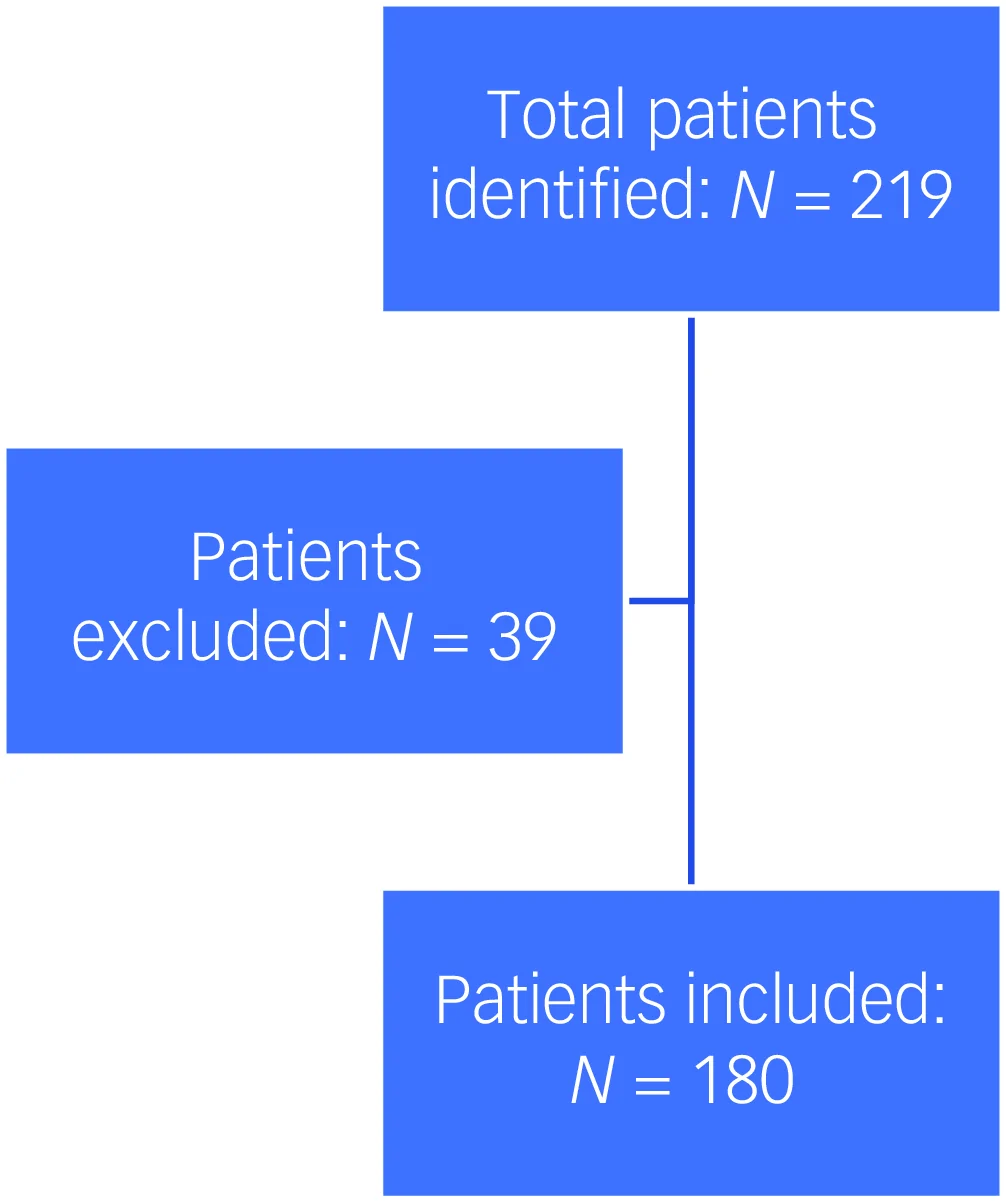
Patients included in the audit.

A total of 219 patients were registered to initiate clozapine during the study period. Complete data were available for 180 patients (82%) (Fig. 1). Patients excluded were those whose medical notes were unavailable and those who opted out of research. The mean age was 44 years (range: 21–85 years). Diagnostic categories included schizophrenia (*n* = 143, 80%), schizoaffective disorder (*n* = 21, 12%), Parkinson’s disease (*n* = 5, 3%) and other diagnoses (*n* = 9, 5%) (Table 1).

**Table 1:** Baseline demographic characteristics Table 1 long description.

Parameters	*N* = 180
Age, years	
Mean	44
s.d.	15.00
Range	21–85
Sex, *n* (%)	
Male	131 (73%)
Female	49 (27%)
Diagnosis, *n* (%)	
Schizophrenia	145 (80%)
Schizoaffective disorder	21 (12%)
Parkinson’s disease	5 (3%)
Other	9 (5%)
Ethnicity, *n* (%)	
White	147 (82%)
Black or Black British	11 (6%)
Asian	12 (7%)
Mixed	6 (3%)
Other	4 (2%)

### Baseline monitoring

**Fig. 2:**
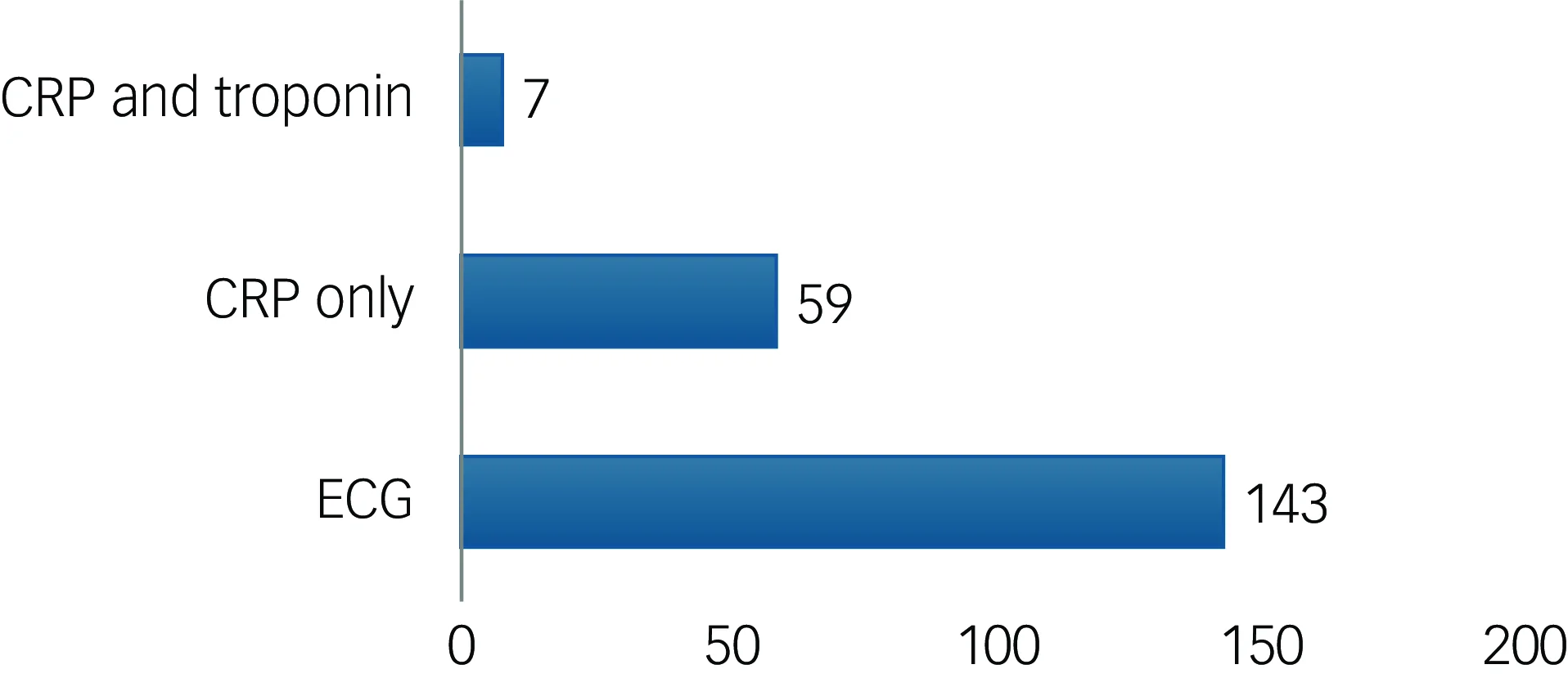
Baseline cardiac monitoring. CRP, C-reactive protein; ECG, electrocardiography.

Baseline ECG was documented in 143 patients (80%), whereas 37 patients (20%) had no ECG recorded before clozapine initiation. By contrast, baseline biomarker monitoring was extremely limited. Only 7 patients (4%) had both troponin and CRP measured before starting clozapine, and 59 patients (33%) had a baseline CRP recorded (Fig. 2).

### Monitoring during first 4 weeks

The first 4 weeks represent the highest-risk period for clozapine-induced myocarditis. However, monitoring during this phase was inconsistent. There was no evidence of routine troponin testing, and CRP measurements were sporadic, offering limited clinical utility. In total, 4% of patients (8 of the 180 with complete data) had troponin and CRP assessed weekly for 4 weeks. A further 25 patients had CRP assessed once in the first 4 weeks. It is unclear whether the rationale for the CRP assessments in these patients was a cardiology recommendation or random events.

### Clinical symptoms during the initial 4 weeks

**Fig. 3:**
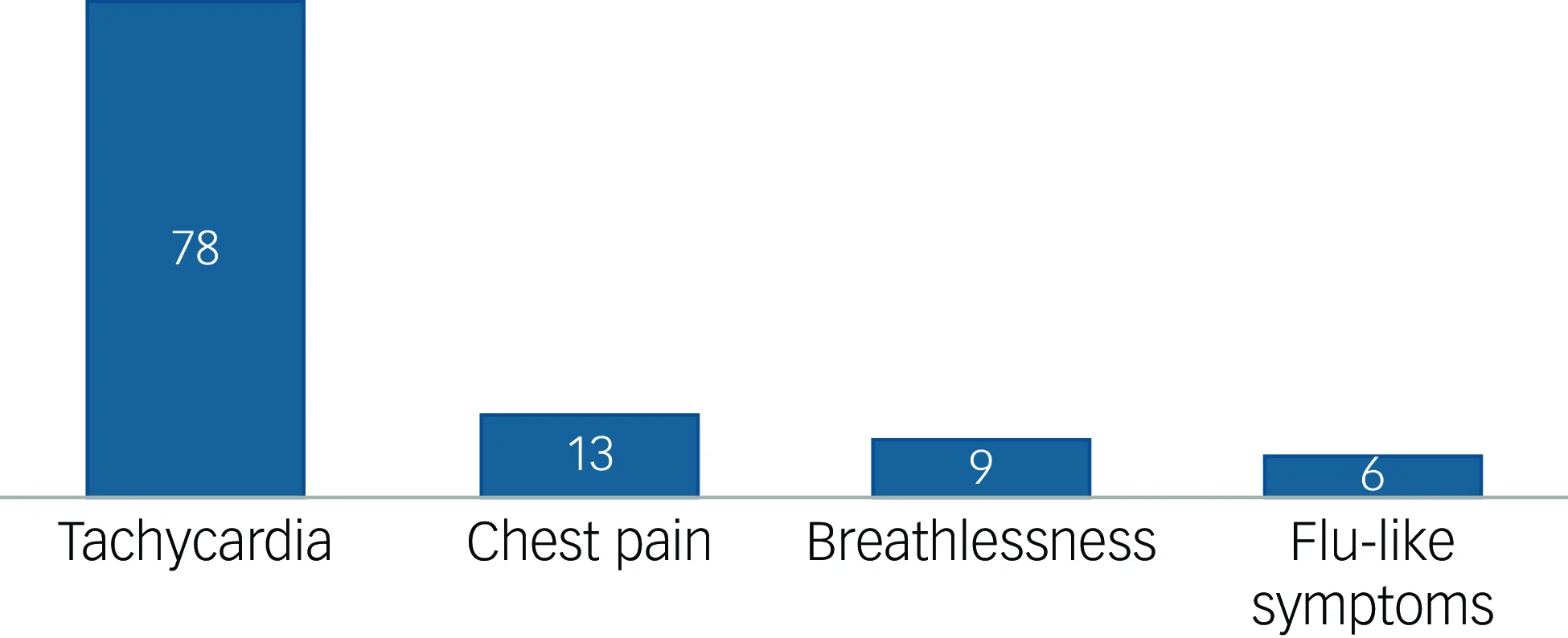
Frequencies of cardiac-related adverse effects during the first 4 weeks of clozapine treatment.

Tachycardia was the most common cardiac side-effect during the initiation phase and was documented in 43% of patients (78 of 180). Other symptoms included chest pain (7%, *n* = 13), breathlessness (5%, *n* = 9) and flu-like symptoms (3%, *n* = 6) (Fig. 3).

### Cardiology referrals

**Fig. 4:**
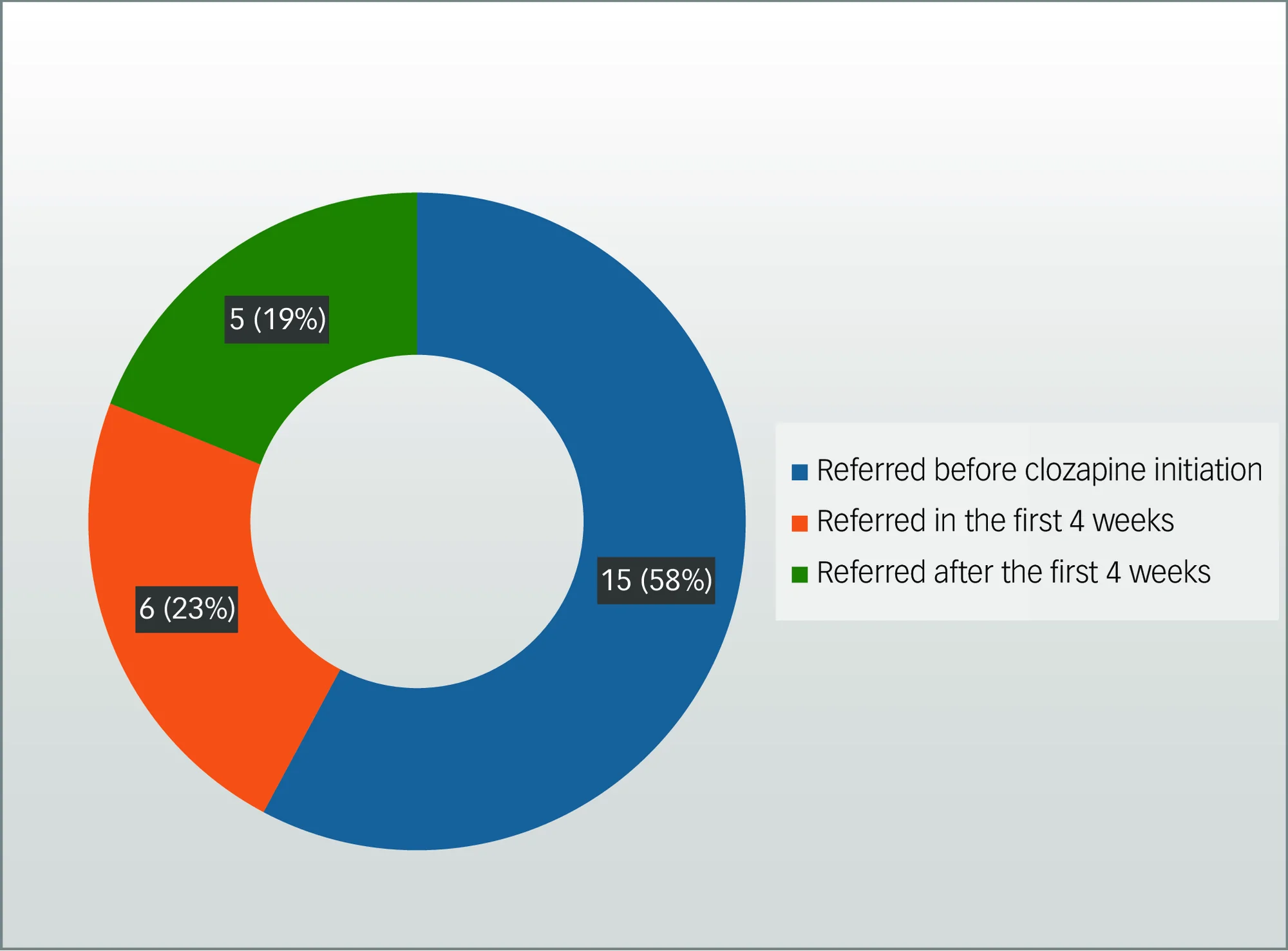
Cardiology referrals before and during clozapine treatment.

Notably, 8% of patients (15 of 180) were referred for cardiology assessment before clozapine initiation. The main reasons for the referrals were ECG abnormalities or a family or personal history of cardiac disease. One patient with Wolff–Parkinson–White syndrome was referred for assessment before clozapine initiation. Cardiology recommendations included echocardiograms, blood tests including CRP and troponin, and rate-controlling medications such as beta-blockers and ivabradine. A further six patients were referred during the first 4 weeks of clozapine titration, mainly owing to tachycardia. Five patients were referred after the initial 4-week titration period (Fig. 4).

### Treatment discontinuation

Clozapine was discontinued owing to suspected cardiac complications in 7% of patients (*n* = 12) during the first 4 weeks of clozapine initiation. Reasons included persistent tachycardia, chest pain and abnormal ECG findings. Two of the seven patients who had baseline and weekly troponin and CRP discontinued clozapine for cardiac reasons within the first 4 weeks. Of the patients involved in the 26 referrals to cardiology before and during clozapine treatment, 4 patients stopped in the first 4 weeks. One patient discontinued after 1 year because of suspected myocarditis.

## Discussion

There is an urgent need to increase the uptake of clozapine in individuals with treatment-resistant schizophrenia. Clozapine is a life-preserving intervention that significantly reduces rates of readmission to hospital, aggression, substance misuse and suicide in this population. It should be initiated promptly once treatment resistance has been established. It is equally important to ensure that clozapine is not discontinued without thorough evaluation of the associated risks and benefits, supported by appropriate monitoring and, where necessary, specialist referral to inform clinical decision-making.

Clozapine was withdrawn globally in the early 1970s when it was found to be responsible for causing agranulocytosis that resulted in the death of eight patients in Finland.^19^ It was subsequently reintroduced following a landmark trial by Kane et al,^20^ with strict haematological monitoring guidelines. Since then, the use of clinical guidelines has been an essential feature in the safe use of clozapine. Although there are well-recognised guidelines for haematological monitoring, the same cannot be said for myocarditis. Indeed, evaluation of clozapine prescribing shows substantial worldwide variation in safety monitoring practices.^21,22^ The significant underuse of clozapine among patients with treatment-resistant schizophrenia is due in part to the stringent monitoring requirements.^23^ However, it is important to note that although excessive regulatory monitoring may be a deterrent to clozapine use, an absence of safeguards may increase patient risk. A balanced and pragmatic approach is required to achieve minimum safety of clozapine prescribing and monitoring standards.

### What are the current clozapine cardiac monitoring guidelines?

In the UK, clozapine manufacturers suggest a baseline ECG. They recognise that the risk of myocarditis is greatest in the first 2 months of treatment and recommend that clozapine should be promptly stopped if myocarditis or cardiomyopathy is suspected. However, monitoring of CRP or troponin is not specifically recommended or mandated. The National Institute for Health and Clinical Excellence schizophrenia treatment guidelines,^24^ albeit very dated, make no recommendation pertaining to cardiac monitoring of clozapine. The Maudsley Prescribing Guidelines (15th edition)^25^ recommend baseline ECG and echocardiography (if available), full blood count, CRP and troponin. In addition, for the first 4 weeks, they recommend weekly CRP and troponin (and, if possible, ECG) in addition to the mandatory weekly full blood count.

In some countries – notably, Australia, New Zealand and Germany – guidelines require mandatory weekly monitoring of troponin and CRP in the first 4–6 weeks of treatment.^26^ A review of the most recent literature found near unanimity on the necessity of cardiac monitoring during clozapine initiation. According to the most recent multidisciplinary consensus statement, minimum cardiac monitoring during clozapine initiation should include baseline and weekly CRP, and troponin for at least 4 weeks, alongside a full blood count and assessment of temperature, oxygen saturation, ECG and postural hypotension.^18^ Other guidelines go a step further recommending evaluation of pro-BNP weekly for 8 weeks in addition to CRP and troponin.^27,28^ The rate of dose escalation during the titration phase and concomitant prescription of valproate is recognised as a risk factor for clozapine-induced myocarditis.^29,30^ To counter this risk, an international adult guideline was developed to make clozapine titrations safer.^31^ It outlines six personalised titration schedules based on genetics and ancestry and other significant factors involved in the pharmacokinetic disposition of clozapine.

The wide variation in the reported incidence of clozapine-induced myocarditis is most likely to reflect variation in prescribing practices (for example, rate of clozapine titration) and clozapine monitoring recommendations. Ample evidence suggests that a slower rate of clozapine titration reduces the incidence of myocarditis and other inflammatory adverse effects.^31,32^ However, overall, there is the spectre of under-recognition of a potentially fatal adverse effect or, on the other hand, discontinuation of clozapine in the presence of common, benign and easily managed adverse effects.

The results of our audit indicated that at baseline before clozapine initiation, 80% of patients had an ECG. One-third of patients (59 of 180) patients had baseline CRP monitoring only, whereas only seven patients had baseline troponin and CRP measurements. In the first 4 weeks of clozapine treatment, 25 patients had CRP monitored once, but only 8 patients had a troponin assessed. A total of 26 cardiology referrals were made, but the time intervals varied. Notably, 15 patients were referred to cardiology before clozapine initiation. Further, 6 patients were referred in the first 4 weeks, and another 5 referrals were made after the initial titration period. It is likely that the cardiology referrals and recommendations account for the troponin assessments at baseline and in the first 4 weeks in patients for whom this occurred.

In the first 4 weeks, a total of 12 patients discontinued clozapine because of cardiac-related adverse effects. We did not evaluate whether clozapine withdrawal was essential or precautionary. Furthermore, we did not assess whether the patients were subsequently rechallenged. Symptoms that may or may not indicate myocarditis, such as tachycardia and fever, occur commonly during clozapine initiation and can lead to premature termination of treatment. Minimum cardiac monitoring including baseline ECG, troponin and CRP assessment, followed by once weekly troponin and CRP for at least 4 weeks, would ensure that myocarditis is promptly detected and appropriate cardiology referrals are undertaken, and that unnecessary clozapine discontinuation is avoided. Given the strategic importance of clozapine in treatment-resistant schizophrenia, having access to prompt cardiology services is crucial, but we acknowledge that this may not always be the case. According to Patel et al,^33^ the cessation of clozapine, while sometimes necessary, is not always mandatory. From a practical perspective, there are well-established medical therapies for left ventricular systolic dysfunction of any cause that can significantly improve cardiac function and may facilitate continued therapy with clozapine.

### Limitations

This audit had several limitations. First, its retrospective design meant that it relied on existing clinical documentation, which may have been incomplete or inconsistently recorded, particularly with respect to symptom reporting and cardiology referrals. Second, data were unavailable for 18% of registered patients, which may have introduced selection bias. Laboratory results for troponin and CRP were sometimes absent or not documented in a standardised format, despite efforts to retrieve data directly from pathology systems. Third, we did not assess whether clozapine discontinuation for suspected cardiac events was clinically justified or precautionary, nor whether patients were subsequently rechallenged. We did not investigate the outcomes of the cardiology referrals. In addition, the audit did not evaluate the clinical impact of monitoring practices, such as whether inadequate surveillance resulted in missed cases of myocarditis or adverse outcomes. Finally, findings reflect practices within a single NHS trust and may not be generalisable to other regions or healthcare systems. Future research should use prospective designs, standardised data collection, and outcome verification to address these gaps.

### Clinical implications

This audit revealed substantial gaps in cardiac monitoring during clozapine initiation within KMMH. Although most patients (80%) received a baseline ECG, monitoring of troponin and CRP was limited both at baseline and during the first 4 weeks, which is the period of highest risk for clozapine-induced myocarditis. Only 4% of patients had baseline CRP and troponin measurements, and CRP monitoring was sporadic, despite evidence supporting these biomarkers as sensitive indicators of early myocardial inflammation. Furthermore, tachycardia was documented in more than 40% of patients and was a common cause of treatment discontinuation, with 7% of patients discontinuing clozapine owing to suspected cardiac complications. Without standardised monitoring, there is a dual risk of under-recognition of myocarditis and unnecessary discontinuation of clozapine, which may compromise treatment outcomes.

Embedding routine cardiac monitoring into clinical practice is both feasible and essential. Incorporation of troponin and CRP monitoring into mandatory weekly full blood count schedules would require no additional blood sampling and would be consistent with international best practice. Adoption of a standardised protocol could improve early detection, reduce morbidity, and support safe and sustained clozapine use. Future work should evaluate the impact of enhanced monitoring on clinical outcomes and explore strategies for harmonisation of national guidelines.

## Data Availability

The data that support the findings of this study are available from the corresponding author (E.W.) upon reasonable request.

## Table 1 Long description

A table summarizing demographic characteristics of 180 patients. The table has 10 rows and 4 columns. The columns are labeled Parameters, and N = 180. The rows include Age in years with Mean 44, standard deviation 15.00, and Range 21-85. Sex is divided into Male with 131 (73 percent) and Female with 49 (27 percent). Diagnosis includes Schizophrenia with 145 (80 percent), Schizoaffective disorder with 21 (12 percent), Parkinson’s disease with 5 (3 percent), and Other with 9 (5 percent). Ethnicity is categorized into White with 147 (82 percent), Black or Black British with 11 (6 percent), Asian with 12 (7 percent), Mixed with 6 (3 percent), and Other with 4 (2 percent).

Navigate back to Table 1.
